# Fibromyalgia Syndrome: Etiology, Pathogenesis, Diagnosis, and Treatment

**DOI:** 10.1155/2012/426130

**Published:** 2012-11-04

**Authors:** Enrico Bellato, Eleonora Marini, Filippo Castoldi, Nicola Barbasetti, Lorenzo Mattei, Davide Edoardo Bonasia, Davide Blonna

**Affiliations:** ^1^Department of Orthopedics and Traumatology, CTO—Maria Adelaide Hospital, University of Turin Medical School, Via Zuretti 29, 10126 Turin, Italy; ^2^Department of Orthopedics and Traumatology, Mauriziano Umberto I Hospital, University of Turin Medical School, Largo Turati 62, 10128 Turin, Italy

## Abstract

Fibromyalgia syndrome is mainly characterized by pain, fatigue, and sleep disruption. The etiology of fibromyalgia is still unclear: if central sensitization is considered to be the main mechanism involved, then many other factors, genetic, immunological, and hormonal, may play an important role. The diagnosis is typically clinical (there are no laboratory abnormalities) and the physician must concentrate on pain and on its features. Additional symptoms (e.g., Raynaud's phenomenon, irritable bowel disease, and heat and cold intolerance) can be associated with this condition. A careful differential diagnosis is mandatory: fibromyalgia is not a diagnosis of exclusion. Since 1990, diagnosis has been principally based on the two major diagnostic criteria defined by the ACR. Recently, new criteria have been proposed. The main goals of the treatment are to alleviate pain, increase restorative sleep, and improve physical function. A multidisciplinary approach is optimal. While most nonsteroidal anti-inflammatory drugs and opioids have limited benefit, an important role is played by antidepressants and neuromodulating antiepileptics: currently duloxetine (NNT for a 30% pain reduction 7.2), milnacipran (NNT 19), and pregabalin (NNT 8.6) are the only drugs approved by the US Food and Drug Administration for the treatment of fibromyalgia. In addition, nonpharmacological treatments should be associated with drug therapy.

## 1. Introduction

Fibromyalgia is a syndrome characterized by chronic widespread pain at multiple tender points, joint stiffness, and systemic symptoms (e.g., mood disorders, fatigue, cognitive dysfunction, and insomnia) [1–4] without a well-defined underlying organic disease. Nevertheless, it can be associated with specific diseases such as rheumatic pathologies, psychiatric or neurological disorders, infections, and diabetes.

What today is defined as fibromyalgia had already been described in the nineteenth century. In 1904, Gowers [5] coined the term “fibrositis” which was used until the seventies and eighties of the last century when an etiology involving the central nervous system was discovered. But it was Graham [6] in 1950 who introduced the modern concept of fibromyalgia as “pain syndrome” in the absence of a specific organic disease. Then in the mid-1970s Smythe and Moldofsky [7] coined the new term “fibromyalgia” and identified regions of extreme tenderness, the so-called “tender points.” Only in 1990 did the American College of Rheumatology committee write up the widely used diagnostic criteria [8] that have only recently been modified [9, 10]. The prevalence of fibromyalgia has been estimated to be around 1%-2% (3.4% for women and 0.5% for men) [11, 12]. However, it is still a poorly understood condition that is difficult to diagnose.

This paper is based on a systematic search of the PubMed database to identify articles related to fibromyalgia. The searches were restricted to English language citations from the inception of the database to June 2012. Relevant articles from the bibliographies of selected articles were also identified and used in this paper. All selected articles were published between 1904 and 2012.

This paper is primarily intended to assist orthopedic surgeons who find themselves faced with patients' referring musculoskeletal symptoms affected by (often undiagnosed) fibromyalgia. It is very important to know and to remember this syndrome so that the patient can be sent to the correct specialist.

## 2. Etiology and Pathogenesis

The etiology and pathogenesis of fibromyalgia are still not fully understood. Several factors such as dysfunction of the central and autonomic nervous systems, neurotransmitters, hormones, immune system, external stressors, psychiatric aspects, and others seem to be involved.

### 2.1. Central Nervous System (CNS)

Central sensitization is considered the main mechanism involved and it is defined by the increased response to stimulation mediated by CNS signaling [13]. Central sensitization is the consequence of spontaneous nerve activity, enlarged receptive fields, and augmented stimulus responses transmitted by primary afferent fibers [14]. An important involved phenomenon seems to be the “windup” which reflects the increased excitability of spinal cord neurons: after a painful stimulus, subsequent stimuli of the same intensity are perceived as stronger [15]; this occurs normally in everyone [16], but it is excessive in fibromyalgic patients [17]. These phenomena are an expression of neuroplasticity and are mainly mediated by N-methyl-D-aspartate (NMDA) receptors located in the postsynaptic membrane in the dorsal horn of the spinal cord [18–21].

Another mechanism supposedly involves the well-known descending inhibitory pain pathways, which modulate spinal cord responses to painful stimuli. They seem to be impaired in patients with fibromyalgia, helping to exacerbate the central sensitization [14, 22, 23].

Apart from augmented neuronal mechanisms, glial cell activation also appears to play an important role in the pathogenesis of fibromyalgia because they help to modulate pain transmission in the spinal cord. Activated by various painful stimuli, they release proinflammatory cytokines, nitric oxide, prostaglandins, and reactive oxygen species that stimulate and prolong spinal cord hyperexcitability [24–26]. 

Also, various neurotransmitters seem to be involved in the central sensitization. Serotonin (5-HT) has a significant role in the modulation of pain [27], and several studies have been carried out looking for modified levels of this molecule in the serum and in the cerebrospinal fluid (CSF). Also, 5-HT's precursor tryptophan and its metabolites have been measured in the blood and in the CSF and the urine with conflicting data. In some studies 5-HT was found at low levels either in the serum [28–30] or in CSF [31] while other authors have not found statistical differences in 5-HT levels between affected patients and controls either in serum [28] or in CSF [32]. Serotonin is involved also in the regulation of mood and sleep [33, 34] and this could explain the association between fibromyalgia and sleep and mental disorders.

Other neurotransmitters also play a role. There are data suggesting the involvement of norepinephrine [32], dopamine [35, 36], substance P (whose levels are typically high in cases of fibromyalgia as its synthesis is inhibited by 5-HT) [37–39], endorphins, and metenkephalins [40, 41]. These peptides of the endogenous opioid system seem to be hyperactive but somehow are unable to modulate pain in these patients. This could explain the reduced efficacy of exogenous opiates in this population [42].

Functional neuroimaging studies support the involvement of the brain in the pathogenesis of this condition.

Single-photon-emission computed tomography (SPECT) was one of the first functional neuroimaging techniques to be used for this. After the infusion of a radioactive tracer, this technique provides a measure of regional cerebral blood flow (rCBF) throughout the brain, reflecting neural activity. The thalamus seems to be a region of interest. Mountz et al. [43] compared baseline levels of rCBF in 10 patients with 7 controls, demonstrating decreased rCBF in bilateral thalamus and caudate nucleus in the first group. The involvement of thalamus and basal ganglia was also proposed by Adigüzel et al. [44]: he demonstrated increases in rCBF in these regions after treatment with amitriptyline. Kwiatek et al. [45] showed decreased rCBF in the right thalamus, the inferior dorsal pons, and next to the right lentiform nucleus. Bradley et al. [46] also replicated the finding of low rCBF in the right thalamus.

Positron emission tomography (PET) uses radioactive tracers like SPECT, but it is characterized by increased temporal and spatial resolution. For example, Wik et al. [47] compared rCBF in 8 patients and controls at rest. Patients showed higher rCBF than controls in the retrosplenial cortex bilaterally, while lower rCBF in the left frontal, temporal, parietal, and occipital cortices. This could reflect increased attention towards subnoxious somatosensory signaling and a dysfunction of the normal cognitive processing of pain in patients affected by fibromyalgia. PET with [_18_F] fluorodeoxyglucose (FDG) helps to assess variations in glucose metabolism. Yunus et al. [48] did not find significant differences at rest between 12 fibromyalgic patients and 7 controls, while Walitt et al. [49] showed an association between the increased metabolic activity in limbic structures and the improvement in a comprehensive treatment program. Using the radiolabeled opioid [_11_C] carfentanil, Harris et al. [40] tried to explain the apparently paradoxical hyperactivity of the endogenous opioid system. He showed a significantly reduced overall *μ* opioid receptor binding potential in patients affected by fibromyalgia. The right and left nucleus accumbens and the left amygdale were the regions most significantly involved and a trend towards reduction was also seen in the right dorsal anterior cingulate cortex. These findings could reflect occupancy by endogenous opioids released in response to the ongoing pain and a receptor downregulation.

Functional magnetic resonance imaging (fMRI) has greater temporal and spatial resolution than either SPECT or PET. In the first fMRI study of fibromyalgia [50] 16 patients and 16 controls underwent fMRI during painful stimulation. First the 2 groups were stimulated with the same pressure; then the intensity of stimulation of controls was increased to deliver a subjective level of pain similar to that experienced by the patients. Neural activation patterns were similar only under the second condition of painful stimulation. These findings support the hypothesis of a model of centrally augmented pain processing. Cook et al. [51] examined with fMRI the response of two groups of women (9 patients and 9 controls) to both painful and nonpainful stimuli. In response to nonpainful stimuli patients showed significantly greater activity than controls in insular, prefrontal, supplemental motor and anterior cingulate cortices. In response to painful stimuli the contralateral insular cortex was significantly activated to a greater extent in patients than it was in controls. Other recent studies have suggested the role of cortical structures, such as the insular cortex [52, 53], the right premotor cortex, the supplementary motor area, the midcingulate cortex, and the right inferior frontal gyrus [52].

The role of the thalamus has been recently underlined by Foerster et al. [54] who explored correlations between rCBF and levels of both clinical and evoked pain. They showed a significant correlation between the rCBF in the bilateral thalami and the BPCQ-INT scale [55], a self-report questionnaire used to evaluate personal control of pain. In addition the rCBF values of the thalamus appeared less correlated with the values detected in other brain regions in patients than in controls.

Functional magnetic resonance imaging seems also to be useful to better understand the role of the descending inhibitory pain system. In the study done by Jensen et al. [56] 16 patients and 16 controls underwent fMRI during individually calibrated painful pressure. They did not find different activities in brain regions involved in attention or affectivity, or regions with sensory connections with the stimulated body area. Interestingly, they showed attenuated response to pain in the rostral anterior cingulate cortex, an important region of the descending pain regulating system. This may explain the impairment of this system, as previously proposed by various authors [14, 22, 23]. In a subsequent study Jensen compared the functional connectivity of the descending inhibitory pain pathways in fibromyalgic patients and controls [57]. He mainly focused on the rostral anterior cingulate cortex and the thalamus. Controls showed higher rACC connectivity to the bilateral hippocampi, amygdala, brainstem, and the rostral ventromedial medulla (regions involved in the pain inhibitory network); they also showed higher thalamus connectivity to the orbitofrontal cortex (the region involved in pain and emotion regulation through both cognitive evaluative processes and homeostatic control). 

Additional neuroimaging methods have been used to understand central pathogenesis of fibromyalgia.

Voxel-based morphometry (VBM) uses MRI images to assess the volume of specific brain regions. Various studies have shown loss of grey matter in fibromyalgic patients involving the amygdala, the cingulate cortex, and the hippocampus [58–61]. The significance of this atrophy is not clear.

Diffusion tensor imaging (DTI) is based on the movement of water through brain tissue and provides information about the integrity of white matter. Sundgren et al. [62] showed a reduced signal in the right thalamus in patients affected by fibromyalgia and the reduction was statistically greater in patients referring worse pain.

Magnetic resonance spectroscopy (MRS) evaluates a signal that reflects the concentration of various molecules (e.g., glycine, glutamate, and aspartate) in relation to a standard molecule. Patients affected by fibromyalgia showed a different ratio of choline/creatine within the dorsolateral prefrontal cortex and a different ratio of glutamate/glutamine within the insula and the posterior gyrus [63–65].

Arterial spin labeling (ASL) is similar to fMRI, but uses magnetized blood as contrast agent. The signal of rCBF is mostly from the parenchyma improving the spatial localization. Few authors have used ASL: Owen found abnormal activations in the bilateral insula, the secondary somatosensory cortex, the cingulate cortex, the contralateral primary somatosensory cortex, and the ipsilateral thalamus [66]; Hernandez found decreased rCBF in the bilateral thalamus in fibromyalgic patients [67].

### 2.2. Neuroendocrine System and Autonomic Nervous System

As fibromyalgia is considered a stress-related disorder, it is easy to understand that the hypothalamic-pituitary-adrenal (HPA) axis is involved [68]. Different studies showed elevated cortisol levels, particularly in the evening, associated with a disrupted circadian rhythm [69, 70]. In addition, these patients showed high values of adrenocorticotropic hormone (ACTH) both basally [71, 72] and in response to stress—most likely as a consequence of a chronic hyposecretion of corticotropin-releasing hormone (CRH) [73]. These alterations are probably related to low levels of 5-HT observed in cases of fibromyalgia, because serotoninergic fibers regulate the HPA axis function [71].

Growth hormone (GH) levels tend to be normal during the day, reduced during sleep. It is likely that the explanation is twofold. First, GH is mainly secreted during stage 4 of sleep and this phase is disrupted in patients affected by fibromyalgia. Second, these patients have high levels of somatostatin, a GH inhibitor, induced by ACTH whose levels are high as previously mentioned [74]. Thyroid hormone levels are usually normal, even if the patients often show symptoms of hypothyroidism and there is some evidence suggesting an association with abnormal thyrotropin-releasing hormone (TRH) stimulation tests [75]. Gonadotropin and gonadal steroid secretion is usually normal [76–78], apparently without any correlation to the higher incidence of fibromyalgia in females.

Various studies, the most recent of which is based on methodologies such as power spectrum analysis of heart rate variability [79, 80] and tilt table tests [81], seem to confirm that in fibromyalgia the sympathetic nervous system is persistently hyperactive, but hyporeactive to stress. This could explain some clinical symptoms such as fatigue, morning stiffness, sleep disorders, anxiety, pseudo-Raynaud's phenomenon, sicca symptoms, and bowel irritability [42]. High serum levels of neuropeptide Y, which is normally secreted along with norepinephrine, are supposed to be a sign of this dysautonomic state [82].

### 2.3. Sleep Disturbances

Patients with fibromyalgia often complain of sleep disorders [83] and these are probably involved in its pathogenesis [1]. As revealed by electroencephalographic examinations, the fourth phase of sleep is the most disturbed and a direct consequence should be a deficit of GH and insulin-like growth factor 1 (IGF-1) [84, 85]. Given that these hormones are involved in muscle microtrauma repair, the healing of this tissue could be affected by sleep disturbances [86].

### 2.4. Genetic Factors

Genetic predisposition is likely to be an important factor as suggested by several familial studies [87, 88] and transmission is thought to be polygenic [89]. Among the various genes investigated, the most important are associated with neurotransmitters. The serotonin transporter gene is characterized by a single nucleotide polymorphism; the “S” (short) allele is more frequent in patients affected by fibromyalgia and by psychological distress [90, 91]. Other genes presumed to be involved are the catechol-O-methyltransferase gene [92, 93], the dopamine D4 receptor gene [94], and the HLA-region [95].

### 2.5. Immune System

Fibromyalgia is common in patients affected by autoimmune disease [96–98]. Different studies in the literature deal with autoantibodies in fibromyalgia [99–102] with equivocal results. In particular several authors have investigated the association between this disease and antipolymer antibodies (APAs) [103, 104]: the results are controversial and APAs cannot be used as a marker for diagnosis [105].

### 2.6. Psychiatric Aspects

Psychiatric problems seem to contribute considerably to the development of fibromyalgia. The prevalence of psychiatric conditions among patients affected by fibromyalgia is higher than among subjects complaining of other rheumatic diseases [3]. The most common disorders associated are anxiety, somatization, dysthymia, panic disorders, posttraumatic stress, and overall depression [106–110]. Depression is more frequently associated with fibromyalgia than with other musculoskeletal diseases [111] and the dysfunction of the 5-HT system might play a role [112]. Depression worsens fibromyalgic symptoms and vice versa, and antidepressants represent a cornerstone of fibromyalgia therapy [113–115].

### 2.7. Peripheral Tissues

Peripheral tissues such as skin, muscles, and microvessels are coming under closer investigation. Vascular dysregulation in muscles [116], inadequate response to oxidative stress [117] exacerbated by the nocturnal fall in saturation [118], increased IL-1 in cutaneous tissues, increased substance P in muscles, and DNA fragmentations of muscle fibers [119] are all suspected to possibly play a role in this condition.

### 2.8. Trigger Factors

Infections seem to be able to induce fibromyalgia even if a direct causal relationship is not documented [120]. In particular, viruses such as HCV, HIV, Coxsackie B, and *Parvovirus* [121–124] and bacteria like *Borrelia* [125, 126] could be involved. An important role dealing with this association might be played by cytokines [127–129] and by glial cells, which, for example, express receptors for bacteria and viruses [130, 131].

Physical trauma [132], vaccinations [120], and chemical substances [133] may also be trigger factors. However, it is worth recalling findings by Greenfield who noticed no precipitant factor in 72% of patients included in his research [134].

## 3. Diagnosis

Many cases of fibromyalgia do not precisely align with a standardized set of diagnostic criteria. However, it is not believed to be a diagnosis of exclusion, although some healthcare providers have labeled it as such. Because there is an absence of absolute, definitive diagnostic criteria with universal applicability, providers often settle upon this diagnosis following negative testing for other differentials [135]. 

Rather than assuming a diagnosis of fibromyalgia, carefully considering a multitude of potential diagnoses (shown in Table 1) will decrease the likelihood of a misdiagnosis. Five of the common differentials to consider in patients exhibiting symptoms of fibromyalgia are mental health disorders, hypothyroidism, rheumatoid arthritis, adrenal dysfunction, and multiple myeloma [136].

Diagnosis is difficult and frequently missed because symptoms are vague and generalized. Despite this, three main symptoms are referred by almost every patient: pain, fatigue, and sleep disturbance [137, 138]. In particular the physician must investigate the features of the pain: it is typically diffuse, multifocal, deep, gnawing, or burning. It often waxes and wanes and is frequently migratory. If this is the case, fibromyalgia should be suspected since often this kind of pain is not the result of inflammation or damage in the area of the region(s) of interest. It is also important to evaluate additional symptoms, which may seem unrelated to fibromyalgia, such as weight fluctuations, morning stiffness, irritable bowel disease, cognitive disturbance, headaches, heat and cold intolerance, irritable bladder syndrome, restless legs, and Raynaud's phenomenon [2].

The musculoskeletal and neurological examinations are normal in fibromyalgia patients [139]. A detectable sign is the presence of the tender points, as explained by the American College of Rheumatology (ACR) [8]. 

They are specific places on the body that are painful when a standard amount of pressure (about 4 kg) is applied. 

With respect to laboratory tests, they should be limited to a complete blood count, routine serum chemistries, thyroid-stimulating hormone (hypothyroidism may have symptoms similar to fibromyalgia or may accompany fibromyalgia) and erythrocyte sedimentation rate and/or C-reactive protein. Typically there are no laboratory abnormalities specifically associated with this condition. ANA (antinuclear antibody) test is quite often ordered and it may be positive, but the rate of a positive test in fibromyalgia patients is the same as normal controls [2, 139].

**Figure 1:**
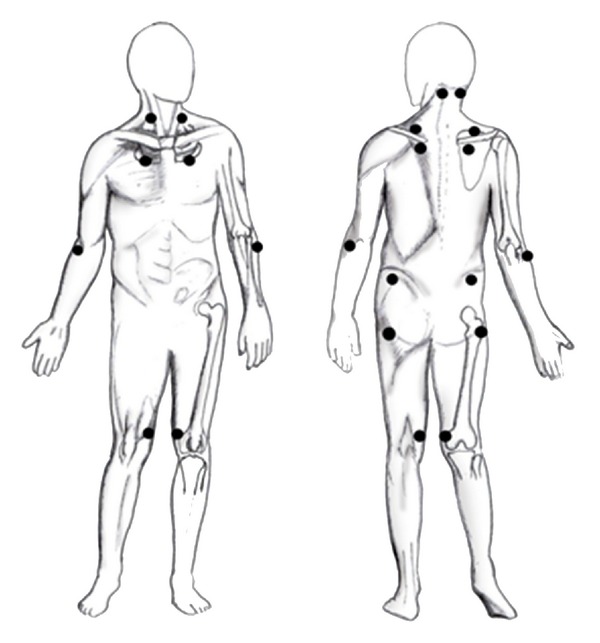
The black dots indicate the 18 tenderness points.

As such, diagnosis is principally based on the two major diagnostic criteria defined by the ACR in 1990 [8]: (1) a history of widespread musculoskeletal pain present for at least three months, and (2) tenderness in at least 11 of 18 defined tender points shown in Figure 1 (both criteria must be satisfied). The pain must affect both sides of the body, must affect areas above and below the waist, and must be also axial. For a tender point to be considered positive it must be evaluated by digital palpation with about 4 kg of pressure (when the thumbnail bed blanches) and the subject must state that the palpation was “painful” (“irritating” is not sufficient). 

A proliferation of studies followed publication of the 1990 criteria. According to PubMed (http://www.ncbi.nlm.nih.gov/pubmed/), 361 English-language original articles with “fibromyalgia” as a keyword were published in the 20 years before the criteria existed, compared with 3844 in the 20-year period after the publication [140]. The 1990 ACR classification criteria have brought numerous benefits: studies have begun to unravel the etiology and impact of the disorder; treatment strategies have improved; patients have also benefited from increased recognition and diagnosis of the disorder. 

Even though these criteria are useful for standardizing the diagnosis, they have been criticized: during these 20 years a number of practical and philosophical objections have been raised in relation to the 1990 ACR classification criteria. The most notable have been the criticisms about the use and interpretation of tender-point count [141, 142], the lack of consideration of associated symptoms [143–146], and neglect of the possibility that fibromyalgia might represent the extreme end of a widespread musculoskeletal pain continuum [147].

To address these issues Wolfe and colleagues undertook a two-phase multicentre study to develop criteria for fibromyalgia that do not require a tender-point count and that provide a severity scale for associated fibromyalgia symptoms: the ACR 2010 criteria [10]. Thirty-two physicians, experienced in fibromyalgia and tender-points examination, interviewed and physically assessed 433 patients with a current or previous diagnosis of fibromyalgia (diagnosed using physicians' usual methods) and 396 matched controls (diagnosed with noninflammatory pain disorders). Two factors best discriminated between patients with fibromyalgia and those with other disorders: the widespread pain index (WPI), a count of number of painful body regions, and the Symptom Severity (SS) scale, a measure of cognitive symptoms, sleep, fatigue, and additional somatic symptoms.

In developing the 2010 ACR criteria, the investigators sought to simplify clinical diagnosis of fibromyalgia, but not to facilitate self-diagnosis; the criteria require a clinical assessment of the severity of comorbid symptoms [10].

The widespread distribution of pain and its chronology remain defining characteristics of fibromyalgia in the 2010 ACR criteria. Importantly, the new criteria also assess the presence and severity of associated symptoms via the SS scale; however, it has been suggested that this new part introduces ambiguity into the clinical diagnosis. For example, there is a notable subjective nature of counting somatic symptoms [148]. How many symptoms constitute “a few,” “a moderate amount,” or “a great deal?” Wolfe and colleagues [10], suggest that the diagnosing physician should make this judgment using their clinical experience to guide them.

This issue has begun to be addressed in the development and modification of the 2010 ACR criteria for use in clinical and epidemiological studies [149].

In their modified 2010 diagnostic criteria (intended for use in postal surveys), Wolfe et al. retain the 19-site WPI and the self-reported specific symptoms, but eliminate the physician estimation of SS score and replace it with three dichotomous “yes/no” answers regarding the presence of abdominal pain, depression, and headaches in the past 6 months [149].

All these criteria are pooled to give an 0–31 fibromyalgia symptoms (FS) score.

The authors report that an FS score of ≥13 correctly classified 93% of patients identified as having fibromyalgia on the basis of the 1990 criteria with a specificity of 96.6% and sensitivity of 91.8% [149].

An implicit aim of the 2010 criteria is to facilitate more rigorous study of fibromyalgia etiology [10]. In common with all complex disorders, the onset of fibromyalgia will be attributable to multiple factors that interact in intricate ways to determine outcome. Studies of etiology need to explore these interactions, although often fail to do so. This problem is neither new nor unique to fibromyalgia research. In epidemiology research, the strongest study design is arguably the prospective cohort study: putative risk factors are measured among a cohort of people without fibromyalgia, who are followed up over time to identify individuals who develop the disorder. The relationship between risk factor exposure and the onset of fibromyalgia can then be assessed, and associations inferred. However, incidence and prevalence of fibromyalgia are low in general population, and the number of disorder-free individuals who would need to be assessed is consequently high.

The 2010 ACR criteria will usefully address the problem, by removing the tender-point examination, and with the publication of modified self-completed criteria that can be included in large scale epidemiological investigations [148]; consequently future studies will be able to assess the large number of people necessary to identify sufficient numbers of incident cases of fibromyalgia.

Wolfe et al. [10] and others [148] have noted that the new criteria are “almost as good” as the previous classification, with the 2010 criteria correctly classifying 80% of subjects who would have been classified using the 1990 criteria. A comparison between 1990 and 2010 ACR classification criteria for fibromyalgia is shown in Table 2.

## 4. Treatment

The goals of fibromyalgia treatment are to alleviate pain, increase restorative sleep, and improve physical function through a reduction in associated symptoms [150]. The identification and treatment of all pain sources that may be present in addition to fibromyalgia such as peripheral inflammatory or neuropathic pain generators (e.g., comorbid osteoarthritis or neuropathic pathologies) or visceral pain (e.g., comorbid irritable bowel syndrome) are central to the proper clinical management of fibromyalgia [151].

Because pain, depression, and other symptoms of fibromyalgia are linked to inherited and environmental causes, a multifaceted treatment approach is often required including both nonpharmacological pain management strategies and medication [152].

The American Pain Society (APS) and the Association of the Scientific Medical Societies in Germany (AWMF) gave the highest level of recommendation to (1) aerobic exercise, (2) cognitive-behavioral therapy (CBT), (3) amitriptyline, and (4) multicomponent therapy. The APS guideline and AWMF guideline were completed prior to the approval of pregabalin and duloxetine for the treatment of fibromyalgia by the United States Food and Drug Administration (FDA). The European League Against Rheumatism (EULAR) gave the highest level of recommendation of “A” to a set of pharmacological treatments (i.e., tramadol, amitriptyline, fluoxetine, duloxetine, milnacipran, moclobemide, pirlindol, tropisetron, pramipexole, and pregabalin), a recommendation strength of “B” to aerobic exercise, and a recommendation strength of only “D” to CBT. EULAR did not give any recommendations for cyclobenzaprine, multicomponent treatment, patient education, hypnotherapy, biofeedback, or other complementary and alternative medicine approaches (CAM), such as acupuncture or homeopathy, whereas EULAR gave a “D” recommendation for CBT, and the APS and AWMF decided on an “A” recommendation. Whereas EULAR and AWMF did not recommend strong opioids (expert opinion), APS recommendation strength was a “C”. APS and AWMF provided the same strength of recommendation (“B”) to tramadol, balneotherapy, hypnotherapy, biofeedback, massage therapy, pregabalin, fluoxetine, and duloxetine. Whereas APS recommended patient education as a single intervention (“B”), acupuncture (“C”), and trigger point injections (“C”), AWMF did not recommend patient education as a single intervention (“A”), acupuncture (“A”) (a minority report recommended acupuncture with a strength of “B”), and trigger point injections (“C”). All three guidelines recommend against the use of NSAIDs (as a single intervention) or corticosteroids [153].

### 4.1. Medications

Evaluating the comparative efficacy of interventions for fibromyalgia is difficult because no common definition of response in fibromyalgia exists. At present, the inclusion of assessment domains is inconsistent, and there is wide variation in the use of instruments indexing those domains. Historically, many symptoms have been thought to be associated with fibromyalgia. Because an assessment of all symptoms in each patient is not feasible; consensus was required to identify the key domains that needed to be assessed to determine clinically meaningful improvement. Much of the work in this area has been organized by the Outcome Measures in Rheumatology (OMERACT) Fibromyalgia working group [154].

It was observed that the responder definitions that best favored drug over placebo included improvement in pain and physical function as well as improvement in either sleep or fatigue (at least 30% improvement over placebo in the symptom domains) [155]. Along with pain, sleep disturbance and fatigue have been consistently ranked by patients and clinicians as being among the most common and troublesome symptoms of fibromyalgia [154]. The other responder definition that performed well in the analysis included additional symptom domains of depression, anxiety, and cognitive dysfunction to reflect the heterogeneity of the fibromyalgia population and the recognition that some treatments may affect these other domains of importance. A responder definition that includes improvement in specific key symptom domains in addition to pain evaluates the broader impact of fibromyalgia on patients and addresses the limitations of other composite responder definitions for fibromyalgia trials that focused only on the symptom of pain.

In terms of nonsteroidal anti-inflammatory drugs (NSAIDs), ibuprofen and naproxen have been shown to be no better than placebo [156], although there is some evidence that NSAIDs may have a synergistic effect when combined with centrally active agents like tricyclic antidepressants and anticonvulsants [157]. Furthermore, a survey of 1042 patients affected by fibromyalgia found that 66.1% deemed NSAIDs more effective than acetaminophen [158]. Given the acceptable adverse effect profile of simple analgesic drugs and, in selected populations, NSAIDS, it seems reasonable to include them in the management of fibromyalgia, despite the lack of conclusive evidence.

The prevalence of opioid use by fibromyalgia patients is unknown, although Goldenberg et al. [159] reported the use of any analgesic drug other than NSAIDs in 52% in a cross-sectional study. Opioids are not, however, recommended by any current guidelines for the management of fibromyalgia [153, 160, 161].

Tramadol has been found to be beneficial in fibromyalgia patients [160, 162]. It is an atypical pain reliever that has a different action on the CNS (the reuptake of serotonin and norepinephrine) from that of other narcotics. Alone or in combination with acetaminophen, it is commonly prescribed at a dose of 200–300 mg/day to relieve fibromyalgia-related pain [163, 164]. Significant differences (*P* ≤ 0.05) were observed between the tramadol/acetaminophen and placebo groups for improvements in sleep adequacy (9.3 versus +6.7) and sleep duration (0.4 hours versus 0.2 hours), but not for the other measures of sleep. Its potential for drug abuse is fortunately negligible, but there is a theoretical risk of seizures and serotoninergic syndrome when it is combined with selective serotonin reuptake inhibitors (SSRIs), serotonin-noradrenalin reuptake inhibitors (SNRIs), monoamine oxidase inhibitors (MAOIs), and triptans, although only a few cases have been described [165].

Both antidepressants and neuromodulating antiepileptics substantially reduce fibromyalgia symptoms. Among antidepressants, serotonin and norepinephrine reuptake inhibitors have been found to provide the best efficacy and tolerability for fibromyalgia [166, 167]. Both duloxetine and milnacipran belong to the serotonin-norepinephrine reuptake inhibitor class of antidepressants and reduce pain by increasing activity of noradrenergic antinociceptive pathways. Both have shown efficacy in randomized, blinded, controlled studies [168].

Duloxetine should be considered in patients with significant depression symptoms. The maximum dosage for the treatment of fibromyalgia is 60 mg daily, but, due to nausea, 30 mg daily is often started initially [169].

Milnacipran has increased selectivity for norepinephrine than for serotonin. It may be helpful in patients with significant fatigue or cognitive dysfunction. The initial dose is 12.5 mg daily which is increased over several weeks to a maximum daily dose of 100 mg, given in two separate doses. In the milnacipran trials, the composite responder definition for the treatment of fibromyalgia consisted of 3 components: (1) 30% improvement from baseline in pain, (2) a rating of “very much improved” (score 1) or “much improved” (score 2) on the Patient Global Impression of Change (PGIC) scale, and (3) six-point improvement from baseline in physical function (SF-36 Physical Component Summary (PCS) score) [170].

Pregabalin is an  *α*2-*δ*  ligand that has analgesic, anxiolytic-like, and anticonvulsant activity in animal models, and biochemical studies have found that the primary binding site for pregabalin and the related gabapentin are  *α*2-*δ*  (type 1).  *α*2-*δ*  is an auxiliary protein associated with voltage-gated calcium channels, and the potent binding of pregabalin at the  *α*2-*δ*  site reduces calcium influx at nerve terminals resulting in reduction of the release of a number of neurochemicals, including glutamate, noradrenaline, and substance P, which may explain the analgesic, anticonvulsant, and anxiolytic-like activity of pregabalin in animal models. It has also been suggested that reducing neurotransmitter release from neurons in the spinal cord and brain may be clinically beneficial for fibromyalgia patients. Pregabalin has significant side effects, including weight gain, dizziness, somnolence, and peripheral edema. When taken with angiotensin-converting-enzyme (ACE) inhibitors it may cause angioedema. The recommended dose is 300–450 mg daily, but many patients respond to lower doses. A single low dose (50–75 mg) at bedtime is frequently employed initially. 

Crofford et al. [171] demonstrated in a 6-month double-blind, placebo-controlled trial that patients treated with pregabalin had statistically significant delayed time to loss of therapeutic response (LTR) versus those receiving placebo. The trial included a 6-week open label (OL) pregabalin-treatment period followed by 26-weeks double-blind treatment with placebo or pregabalin. Adults with fibromyalgia and ≥40 mm score on 100 mm pain visual analog scale (VAS) were considered. During OL weeks 1–3 patients received escalating dosage of pregabalin to determine their optimal dosage. During OL weeks 4–6, patients received their pregabalin optimal fixed dosages (300, 450, and 600 mg/d). To be randomized, they must have had ≥50% decrease in pain VAS and a self-rating of “much” or “very much” improved on Patient Global Impression of Change (PGIC) at the end of OL. Crofford et al. defined the time to LTR as <30% reduction in pain or worsening of fibromyalgia. At the end of double-blind phase, 61% placebo patients met LTR criteria versus 32% pregabalin patients. Similarly, half of placebo patients showed worsening in the Overall Sleep Problem Index of the MOS-Sleep Scale by day 14 compared with by day 42 for pregabalin patients. Regarding adverse events (AE), more pregabalin than placebo patients discontinued the study during the double-blind phase. The most common AEs in the pregabalin treatment group were insomnia (6%), sinusitis, nausea, arthralgia, anxiety, and influenza (each 5%) and weight increased (4%).

Currently duloxetine (DLX), milnacipran (MLN), and pregabalin (PGB) are the only drugs that have been approved by the US Food and Drug Administration (FDA) for the treatment of fibromyalgia. A comparison of the efficacy and harms of these three drugs shows some differences: the NNTs for a 30% pain reduction (all dosages pooled together) were as follows: DLX 7.2 (95% CI 5.2, 11.4), MLN 19 (95% CI 7.4, 20.5), and PGB 8.6 (95% CI 6.4, 12.9). The NNTs for dropout due to lack of efficacy were as follows: DLX −16.5 (95% CI −43.7, −10.1), MLN −31.1 (95% CI −23.7, −16.7), and PGB −16.0 (95% CI −25.8, −11.6). The NNHs for a dropout due to side effects were as follows: DLX 14.9 (95% CI 9.1, 41.4), MLN 7.6 (95% CI 6.2, 9.9), and PGB 7.6 (95% CI 6.3, 9.4) [172].

The mechanisms of action of pregabalin, duloxetine, and milnacipran are thought to be related to proposed pathophysiologies of fibromyalgia. However, these therapeutic agents are still not effective for all fibromyalgia patients. A recent study by Katz et al. suggests that the diagnostic criteria for fibromyalgia may be partially responsible, as there is currently no gold standard for fibromyalgia diagnosis [173].

The family of tricyclic antidepressants (TCAs) are effective over the short term in the management of fibromyalgia, specifically the TCA amitriptyline and the biologically similar cyclobenzaprine. By inhibiting the reuptake of both serotonin and norepinephrine, tricyclic compounds enhance norepinephrine and serotonin neurotransmission in the descending inhibitory pain pathways, resulting in a reduction in pain. Four meta-analyses examined the efficacy of tricyclics in fibromyalgia management [113, 174–176]. These tricyclic studies primarily assessed amitriptyline or cyclobenzaprine in patients with fibromyalgia and tended to be small, short-term, single-center trials. The meta-analysis by Arnold and associates [113] examined 9 placebo-controlled trials of amitriptyline, dothiepin, cyclobenzaprine, clomipramine, and maprotiline. The largest improvement was associated with measures of sleep quality, with treatment effect sizes ranging from 0.10 to 1.19; the most modest improvements were found in measures of stiffness (effect size range, 0.30 to 0.77) and tenderness (effect size range, −0.34 to 0.73). The overall degree of efficacy was modest in most studies, with a median treatment effect size of 0.44 and weighted mean treatment effect size of 0.43. The NNT of amitriptyline is 3,54 (95% CI 2,74, 5,01). Although TCAs are moderately effective, the use of these compounds is limited by a relatively narrow therapeutic index and poor tolerability due to affinity at multiple receptor systems [4]. Unlike newer dual reuptake inhibitors of serotonin and norepinephrine, TCAs possess significant affinity for histaminergic, cholinergic, and adrenergic receptor systems [177, 178], which contribute to their strong side effects such as sedation, dry mouth, and constipation at higher doses. Tolerability of tricyclic compounds can be improved by prescribing very low doses before bedtime and by very slowly escalating the dose. However, the dose should be kept as low as possible, and they should be used with caution in patients with cardiovascular, renal, or hepatic disease [178, 179].

### 4.2. Nonpharmacologic Treatments

The nonpharmacologic treatments most consistently linked to fibromyalgia improvements are aerobic exercise and strength training. Effective exercise focuses on stretching, with gradual progression to strengthening and reconditioning exercise [180, 181].

Two randomized controlled single blind trials indicate that Tai chi holds potential as a useful modality in the multidimensional treatment of fibromyalgia [182]. Tai chi compared to wellness education and stretching improves symptoms, physical function, quality of sleep, self-efficacy, and functional mobility for people with fibromyalgia [183]. Authors observed significant improvements in static balance, dynamic balance, and timed get-up-and-go. These consistent findings of improvement in objective measures of functional mobility carry important clinical implications, suggesting that Tai chi may help decrease risk for falls and minimize difficulties in performing essential daily physical activity tasks [184].

Both aquatic exercises and balneotherapy are regarded as nonpharmacological interventions for fibromyalgia. Aquatic exercises (water-based exercises, aquatic therapy, or hydrotherapy) are exercises that are performed in the water. The Chartered Society of Physiotherapists defined aquatic exercises as a therapy program using the properties of water, designed by a suitably qualified physiotherapist, to improve function, ideally in a purpose-built and suitably heated pool [185]. It remains unclear whether aquatic exercises are more effective than other active interventions such as land-based exercises. Furthermore there is a lack of evidence for specific doses and timing of exercise programs because most RCTs and SRs did not provide enough information to address these issues. Often the intervention was rather poorly described in the original papers [186]. The term balneotherapy (seated immersion or spatherapy) is classically used in (eastern) European countries for bathing in water without exercise. Often natural mineral or thermal waters are used for bathing, drinking, and inhalation. The mechanisms by which immersion in mineral or thermal water or the application of mud alleviates chronic pain and the symptoms of fibromyalgia are not completely known [187–189]. A distinction can be made between the non-specific (hydrotherapeutic in a broad sense) mechanisms of simple bathing in hot tap water and the specific (hydromineral and chemotherapeutic) mechanisms, which depend on the chemical and physical properties of the water used. Hot stimuli produce analgesia on nerve endings by increasing the pain threshold. It causes relief of muscle spasms through the  *γ*  fibers of muscle spindles and activates the descending pain inhibitory system. According to the “gate theory,” pain relief may be due to the temperature and hydrostatic pressure of water on the skin [190].

Spa therapy provokes a series of endocrine reactions, particularly in the release of adrenocorticotropic hormone (ACTH), cortisol, prolactin, and growth hormone (GH), although it does not alter the circadian rhythm of these hormones. A dysregulation of the hypothalamic-pituitary-adrenal (HPA) axis, marked by mild hypocortisolemia and glucocorticoid feedback resistance, has been demonstrated in fibromyalgia patients. These findings can explain the beneficial clinical effects of spa therapy in fibromyalgia. The systematic reviews on patients with fibromyalgia concluded, based on 4 RCTs, that there is moderate evidence in favor of the use of balneotherapy [191]. Unfortunately, no meta-analysis was performed, no data were presented, and most studies showed major methodological flaws.

Psychological pain management skills have also been reported to be efficacious in patients with fibromyalgia. Cognitive-behavioral therapy (CBT) outperformed other psychological treatments in short-term fibromyalgia pain intensity reduction, reaching a medium effect size. Additionally, CBT and relaxation were significantly more effective than other psychological treatments in reducing sleep problems associated with fibromyalgia. The results indicate that all psychological treatments were equally effective in decreasing depression. For pain intensity and depression, the results also indicate that psychological treatments were more effective than control conditions, with small to medium effect sizes [192–194].

Although not included in the recent evidence-based guidelines, EULAR recommendations additionally support inclusion of warm-water therapy, based on consistently beneficial data reported in numerous studies. Long-term studies found that warm-water exercise for 8 months was cost effective, with improvements of 8% for pain and 20% for physical function in treated patients compared with controls [171, 195–197].

Over the last decade, it has been repeatedly shown that noninvasive repetitive transcranial magnetic stimulation (rTMS) of the primary motor cortex (M1) induces analgesic effects both in experimental pain [198–202] and in various chronic pain conditions [203, 204], probably by activating pain modulation systems. Recently it was demonstrated that 10 daily sessions of unilateral M1 stimulation decrease chronic widespread pain and improve health-related quality of life of patients with fibromyalgia [204]. The analgesic effects of rTMS of the primary motor cortex can be maintained for up to 6 months in patients with chronic pain; the decrease in pain intensity was associated with a long-term improvement in other clinical features including fatigue, catastrophizing, and several items related to quality of life. Only a few studies in patients with neuropathic pain [205, 206] or fibromyalgia [204] have evaluated the effects of repeated daily stimulations over a period of 5–10 days and reported analgesic effects lasting for 2-3 weeks after the last stimulation. The mechanisms underlying motor cortex rTMS-induced analgesia remain unclear, but may be similar to that of chronic motor cortex stimulation through surgically implanted epidural electrodes, which is used to treat patients with refractory neuropathic pain [207–209]. Mhalla et al. [210] showed that active rTMS had a significant effect on average pain intensity over the course of the treatment, as shown by comparison with a sham stimulation treatment (*F* = 0.02; *P* = 0.007). Pairwise comparisons showed that this effect was significant from day 5 onwards and was maintained until week 25, although the magnitude of the effect tended to decrease during the period of monthly stimulation, from week 16 to 25. Active stimulation significantly improved (*F* = 8.62, *P* = 0.005); the Brief Pain Inventory (BPI) score reported a marked decrease in the interference of pain with “general activity,” “walking,” “relations with other people,” “enjoyment of life,” and “sleep.” In contrast, the active treatment did not significantly decrease the interference of pain on “work” and “mood.” In addition, active rTMS significantly decreased both the total score (*F* = 5.03; *P* = 0.03) of the Fibromyalgia Impact Questionnaire (FIQ) and the 3 subscores relating to fatigue (*F* = 4.8; *P* = 0.003), stiffness (*F* = 11.7; *P* = 0.001), and morning tiredness (*F* = 7.47; *P* = 0.009).

Mean depression and anxiety scores (Hospital Anxiety and Depression Scale (HAD) and the 13-item short form of the Beck Depression Inventory (BDI)) were not significantly affected by active or sham stimulation. Catastrophizing score (PCS) was significantly lower (*F* = 5.99, *P* = 0.02) after active rTMS than after sham treatment. APR, AWMF and EULAR recommendations are summarized in Table 3.

## 5. Conclusions

Fibromyalgia is a complex syndrome that is often difficult to diagnose, particularly for physicians who do not usually deal with this disease. Pathogenesis is still not fully clear, but modern functional neuroimaging techniques are giving us important data about the CNS involvement. Fibromyalgia is not to be considered a diagnosis of exclusion: the recently published ACR 2010 criteria try to help us not to be confused by all the differential diagnoses for fibromyalgia. A multidisciplinary approach is optimal and the physician must take into consideration both drugs (in particular antidepressants and neuromodulating antiepileptics) and nonpharmacological treatment, such as aerobic exercise and strength training, aquatic exercises and balneotherapy, cognitive-behavioral therapy, and also the emerging brain stimulation techniques. 

## Figures and Tables

**Table 1:** Differential diagnoses for fibromyalgia and corresponding diagnostic testing options.

Differential diagnoses	Diagnostic testing options
Adrenal dysfunction	Morning serum cortisol, urinary catecholamine metabolites
Anemia	CBC with differential, RBC indices (MCV, MHC, MCHC)
Bone marrow disease	WBC differential, ESR, CRP, CMP
Chronic fatigue syndrome	Clinical history
Functional disorders (e.g., intestinal dysbiosis, subtle endocrine imbalances, and postviral immune suppression)	Standard laboratory testing yields unclear results
Hypothyroidism	Thyroid function tests (T3, T4, TSH)
Lyme disease	Lyme titer, CMP
Psychiatric conditions (e.g., PTSD, anxiety, and depression)	Refer to DSM-IV
Multiple sclerosis	MRI scan, lumbar puncture, evoked potential testing
Phenomenological referred myofascial pain	Muscular tender points on physical examination
Rheumatoid autoimmune disorders (e.g., rheumatoid arthritis, ankylosing spondylitis, and scleroderma)	Rheumatic profile (rheumatoid factor, ESR/CRP), ANA
Sleep disorders	EEG sleep studies
Spinal facet pain or sacroiliac joint pain	Radiologic studies (MRI scan, CT scan), bone scans (minimal diagnostic assistance)
Spinal disc herniation	MRI scan
Systemic inflammation or infection	Radiologic studies (MRI scan, CT scan), bone scans (minimal diagnostic assistance)
Vitamin and/or mineral deficiency	Radiologic studies (MRI scan, CT scan), bone scans (minimal diagnostic assistance)

CBC: complete blood count; RBC: red blood cell; MCV: mean corpuscular volume; MCH: mean corpuscular haemoglobin; MCHC: mean corpuscular haemoglobin concentration; WBC: white blood cell; ESR: erythrocyte sedimentation rate; CRP: C-reactive protein; CMP: complete metabolic profile; T3: triiodothyronine; T4: thyroxine; TSH: thyroid-stimulating hormone; PTSD: posttraumatic stress disorder; DSM-IV: diagnostic and statistical manual of mental disorders; ANA: antinuclear antibody; EEG: electroencephalography; MRI: magnetic resonance imaging; CT: computed tomography.

**Table 2:** Comparison between 1990 and 2010 ACR classification criteria for fibromyalgia.

Key features of the ACR 1990 classification criteria for fibromyalgia	ACR 2010 and modified classification criteria for fibromyalgia
*Widespread pain* Pain in the left/right side of the body, pain above/below the waist. In addition, axial skeleton pain (cervical spine or anterior chest or thoracic spine or low back) must be present. *Tender points* Pain, on digital palpation (4 Kg/cm² applied over 4 seconds), must be present in at least 11 of the following 18 specified tender-point bilateral sites: occiput, low cervical, trapezius, supraspinatus, second rib, lateral epicondyle, gluteal, greater trochanter, and knee. *Diagnosis* Both criteria must be satisfied. Widespread pain must be present for at least 3 months. The presence of a second clinical disorder does not exclude the diagnosis of fibromyalgia. * *	*Widespread pain index (WPI)* Note the number of areas in which the patient has had pain over the past week (0–19 points). The following are the areas to be considered: shoulder girdle, hip (buttock, trochanter), jaw, upper back, lower back, upper arm, upper leg, chest, neck, abdomen, lower arm, and lower leg (all these areas should be considered bilaterally). *SS scale score* Fatigue, waking unrefreshed, cognitive symptoms (e.g., working memory capacity, recognition memory, verbal knowledge, anxiety, and depression) [211]. For each of these 3 symptoms, indicate the level of severity over the past week using the following scale: 0 = no problem 1 = slight or mild problems, generally mild or intermittent 2 = moderate; considerable problems, often present and/or at a moderate level 3 = severe; pervasive, continuous, life-disturbing problems Considering somatic symptoms in general, indicate wheter the patient has the following: 0 = no symptoms 1 = few symptoms 2 = a moderate number of symptoms 3 = a great deal of symptoms Final score between 0 and 12 *Criteria* A patient satisfies diagnostic criteria for fibromyalgia if the following 3 conditions are met: (i) WPI ≥ 7/19 and SS scale score ≥ 5 or WPI 3–6 and SS scale score ≥ 9 (ii) symptoms have been present as a similar level for at least 3 months (iii) the patient does not have a disorder that would otherwise explain the pain *Modified criteria* (i) WPI (as above) (ii) SS scale score (as above, but without extent of somatic symptoms) (iii) presence of abdominal pain, depression, headaches (yes = 1, no = 0) The number of pain sites (WPI), the SS scale score, and the presence of associated symptoms are summed to give a final score between 0 and 31

**Table 3:** Comparison between American Pain Society (APS) and Association of the Scientific Medical Societies in Germany (AWMF) with European League Against Rheumatism (EULAR).

	Nonpharmacologic treatment	Medications
APS (American Pain Society) and AWMF (Association of the Scientific Medical Societies in Germany)	*Strong evidence*: Patient education CBT Aerobic exercise Multidisciplinary therapy	*Strong evidence*: Amitriptyline (25/50 mg) NNT 3,54 (95% CI 2,74, 5,01) Cyclobenzaprine (10/30 mg)
	*Moderate evidence*: Strength training Acupuncture Hypnotherapy Biofeedback Balneotherapy	*Moderate evidence*: SNRIs: Milnacipran (100 mg) NNT 7.2 (95% CI 5.2, 11.4) NNH 7.6 (95% CI 6.2, 9.9) Duloxetine (60/120 mg) NNT 19 (95% CI 7.4, 20.5) NNH 14.9 (95% CI 9.1, 41.4) SSRI: Fluoxetine (20/80 mg) Tramadol (200/300 mg) Anticonvulsant: Pregabalin (300/450 mg) NNT 8.6 (95% CI 6.4, 12.9) NNH 7.6 (95% CI 6.3, 9.4)

EULAR (European League Against Rheumatism)	Balneotherapy (grade B)	Tramadol (grade A)
	Individually tailored exercise including aerobic and strength training (grade C)	Analgesics (paracetamol/acetaminophen, weak opioids) (grade D)
	Cognitive-behavioral therapy (grade B)	Antidepressants (amitriptyline, fluoxetine, duloxetine, milnacipran, moclobemide, pirlindol) (grade A)
	Others: relaxation, rehabilitation, physiotherapy, and/or psychological support (grade C)	Tropisetron, pramipexole, pregabalin (grade A)
