# Erratum to “Fibromyalgia Syndrome: Etiology, Pathogenesis, Diagnosis, and Treatment”

**DOI:** 10.1155/2013/960270

**Published:** 2013-01-27

**Authors:** Enrico Bellato, Eleonora Marini, Filippo Castoldi, Nicola Barbasetti, Lorenzo Mattei, Davide Edoardo Bonasia, Davide Blonna

**Affiliations:** ^1^Department of Orthopedics and Traumatology, Mauriziano Umberto I Hospital, University of Turin Medical School, Largo Turati 62, 10128 Turin, Italy; ^2^Department of Orthopedics and Traumatology, CTO-Maria Adelaide Hospital, University of Turin Medical School, Via Zuretti 29, 10126 Turin, Italy

In this paper which appeared in *Pain Research and Treatment* (Volume 2012, Article ID 426130) the affiliations have been switched. The affiliations should appear as shown above.

Moreover in Table 3 on page 10 the NNTs of Milnacipran and of Duloxetine have been switched. The NNTs should appear as follows: Milnacipran NNT 19 (95% CI 7.4, 20.5) and Duloxetine NNT 7.2 (95% CI 5.2, 11.4). The corrected full Table 3 is shown later. 

## Figures and Tables

**Table 3:** Comparison between American Pain Society (APS) and Association of the Scientific Medical Societies in Germany (AWMF) with European League Against Rheumatism (EULAR).

	Nonpharmacologic treatment	Medications
American Pain Society (APS) and Association of the Scientific Medical Societies in Germany (AWMF)	*Strong evidence*:	*Strong evidence*:
	Patient education CBT Aerobic exercise Multidisciplinary therapy	Amitriptyline (25/50 mg) NNT 3,54 (95% CI 2.74, 5.01) Cyclobenzaprine (10/30 mg)
	*Moderate evidence*:	*Moderate evidence*:
	Strength training Acupuncture Hypnotherapy Biofeedback Balneotherapy	SNRIs: Milnacipran (100 mg) NNT 19 (95% CI 7.4, 20.5) NNH 7.6 (95% CI 6.2, 9.9) Duloxetine (60/120 mg) NNT 7.2 (95% CI 5.2, 11.4) NNH 14.9 (95% CI 9.1, 41.4) SSRI: Fluoxetine (20/80 mg) Tramadol (200/300 mg) Anticonvulsant: Pregabalin (300/450 mg) NNT 8.6 (95% CI 6.4, 12.9) NNH 7.6 (95%CI 6.3, 9.4)

European League Against Rheumatism (EULAR)	Balneotherapy (grade B) Individually tailored exercise including aerobic and strength training (grade C) Cognitive-behavioral therapy (grade B) Others: relaxation, rehabilitation, physiotherapy, and/or psychological support (grade C)	Tramadol (grade A) Analgesics (paracetamol/acetaminophen, weak opioids) (grade D) Antidepressants (amitriptyline, fluoxetine, duloxetine, milnacipran, moclobemide, pirlindole) (grade A) Tropisetron, pramipexole, pregabalin (grade A)

